# Characterising the Transmission Dynamics of *Acinetobacter baumannii* in Intensive Care Units Using Hidden Markov Models

**DOI:** 10.1371/journal.pone.0132037

**Published:** 2015-07-01

**Authors:** Tan N. Doan, David C. M. Kong, Caroline Marshall, Carl M. J. Kirkpatrick, Emma S. McBryde

**Affiliations:** 1 Centre for Medicine Use and Safety, Faculty of Pharmacy and Pharmaceutical Sciences, Monash University, Melbourne, Victoria, Australia; 2 Victorian Infectious Diseases Service, The Peter Doherty Institute for Infection and Immunity, Melbourne, Victoria, Australia; 3 Department of Medicine, University of Melbourne, Melbourne, Victoria, Australia; Tianjin University, CHINA

## Abstract

Little is known about the transmission dynamics of *Acinetobacter baumannii* in hospitals, despite such information being critical for designing effective infection control measures. In the absence of comprehensive epidemiological data, mathematical modelling is an attractive approach to understanding transmission process. The statistical challenge in estimating transmission parameters from infection data arises from the fact that most patients are colonised asymptomatically and therefore the transmission process is not fully observed. Hidden Markov models (HMMs) can overcome this problem. We developed a continuous-time structured HMM to characterise the transmission dynamics, and to quantify the relative importance of different acquisition sources of *A*. *baumannii* in intensive care units (ICUs) in three hospitals in Melbourne, Australia. The hidden states were the total number of patients colonised with *A*. *baumannii* (both detected and undetected). The model input was monthly incidence data of the number of detected colonised patients (observations). A Bayesian framework with Markov chain Monte Carlo algorithm was used for parameter estimations. We estimated that 96–98% of acquisition in Hospital 1 and 3 was due to cross-transmission between patients; whereas most colonisation in Hospital 2 was due to other sources (sporadic acquisition). On average, it takes 20 and 31 days for each susceptible individual in Hospital 1 and Hospital 3 to become colonised as a result of cross-transmission, respectively; whereas it takes 17 days to observe one new colonisation from sporadic acquisition in Hospital 2. The basic reproduction ratio (*R*_0_) for Hospital 1, 2 and 3 was 1.5, 0.02 and 1.6, respectively. Our study is the first to characterise the transmission dynamics of *A*. *baumannii* using mathematical modelling. We showed that HMMs can be applied to sparse hospital infection data to estimate transmission parameters despite unobserved events and imperfect detection of the organism. Our results highlight the need to optimise infection control in ICUs.

## Introduction

*Acinetobacter baumannii* is an important pathogen that can cause serious infections such as pneumonia, bacteraemia and meningitis in hospitals, particularly in intensive care units (ICUs) [1]. It is intrinsically resistant to many antibiotics and has a remarkable ability to develop and transmit novel mechanisms of resistance, making treatment extremely difficult with limited therapeutic options available [2]. Infections caused by *A*. *baumannii* are associated with significant mortality (attributable mortality rates ranging from 20% to 37%) and an increase in the average length of ICU stay by 15 days [3–6]. In Australia, the prevalence of *A*. *baumannii* in ICUs is estimated to range from 4% to 20% [7]; whereas the numbers reported in Asia, Europe and North America are 19%, 23% and 4%, respectively [8]. Despite numerous efforts to prevent the spread of healthcare associated infections, the rates of *A*. *baumannii* infections continue to increase worldwide [9–11]. This, in part, is due to an incomplete understanding of the transmission dynamics of this pathogen, which is complex and involves various interrelated factors such as patients, healthcare workers and the hospital environment [12]. In hospitals, acquisition of *A*. *baumannii* is believed to occur by two distinct routes. Acquisition can occur as a result of transmission between patients treated in the ward, mainly via the transiently contaminated hands of healthcare workers (i.e. cross-transmission acquisition) [13]. Alternatively, acquisition may result from sources independent of cross-transmission (i.e. sporadic acquisition) such as colonisation already present at admission and *de novo* colonisation from patient’s gastrointestinal flora [13–15].

Quantifying the relative importance of different acquisition routes is essential for setting infection control priorities [13]. However, quantitative data on the transmission dynamics of *A*. *baumannii* are currently lacking. Such information can be obtained by conducting extensive epidemiological surveillance in combination with genotyping. However, these methods are time-consuming, laborious and may be prohibitively expensive. Additionally, these methods only provide individual patient-level data, and thus are not able to fully capture the complexities and dynamic interactions that determine the spread of the pathogen [16]. Mathematical modelling, by providing a theoretical framework to conceptualise the dynamic interactions between interdependent variables, can overcome these problems. This approach has been used to model the transmission dynamics of Gram-positive organisms in hospitals [17–19]. At present, there are no models specifically developed to examine the transmission dynamics of Gram-negative bacteria including *A*. *baumannii*. In a recent review, we highlighted a need to understand the transmission dynamics of this pathogen using mathematical modelling because such information is of great value for designing effective infection control interventions [20].

Estimating transmission parameters using hospital infection data have a number of statistical challenges. The majority of patients colonised with *A*. *baumannii* carry them asymptomatically [21]. Consequently, the underlying transmission process can only be partially observed and the exact time of acquisition is typically unknown in the absence of frequent routine swabs [18]. Algorithms for data analysis based on hidden Markov models (HMMs) have been shown to be useful for making inferences about an unobserved event and estimating transmission parameters [13,14,22]. In this study, we developed a continuous-time structured HMM to estimate the rates of cross-transmission and sporadic acquisition, and to determine the proportion of *A*. *baumannii* colonisation that was due to these two acquisition sources in the ICU setting in Australia. This HMM framework allowed for imputation of unobserved transmission process using only sparse data on the number of detected colonised patients.

## Methods

### Study setting and data

The study setting was ICUs within three major tertiary hospitals in Melbourne, Victoria, Australia. For the purposes of protecting the identity of the individual hospitals, they have been relabelled Hospital 1, Hospital 2 and Hospital 3 in the present study. The number of ICU beds in the three hospitals was 24, 13 and 32, respectively. At all three hospitals during the study period (January 2000 to December 2004), standard precautions (hand hygiene, cleaning of environment and equipment, use of gowns and gloves according to risk of body fluid exposure, and safe handling and disposal of sharps) were used for patients colonised with susceptible *A*. *baumannii* isolates; whereas contact precautions (use of gowns and gloves for all patient contacts) were used in addition to standard precautions for those colonised with isolates resistant to gentamicin or imipenem. No further infection control interventions were introduced in Hospital 2; whereas two infection control liaison nurses were employed in the ICU in Hospital 1 by the end of the study period. Various infection control measures were introduced in the ICU in Hospital 3 including passive surveillance and feedback, increased environmental cleaning, gowns and gloves for staff, revised antibiotic protocol, and increased infectious diseases physician input. No active screening was performed at the three hospitals. *A*. *baumannii* colonisation was identified by clinical specimens and recorded in an electronic database. The date of ICU admission and discharge was also recorded. Genotype data were not available. This study was approved by the Human Research Ethics Committee at each hospital, and has been comprehensively described previously [7]. Written informed consent was not required; and patient data were anonymised, de-identified and pooled prior to analysis [7].

**Fig 1:**
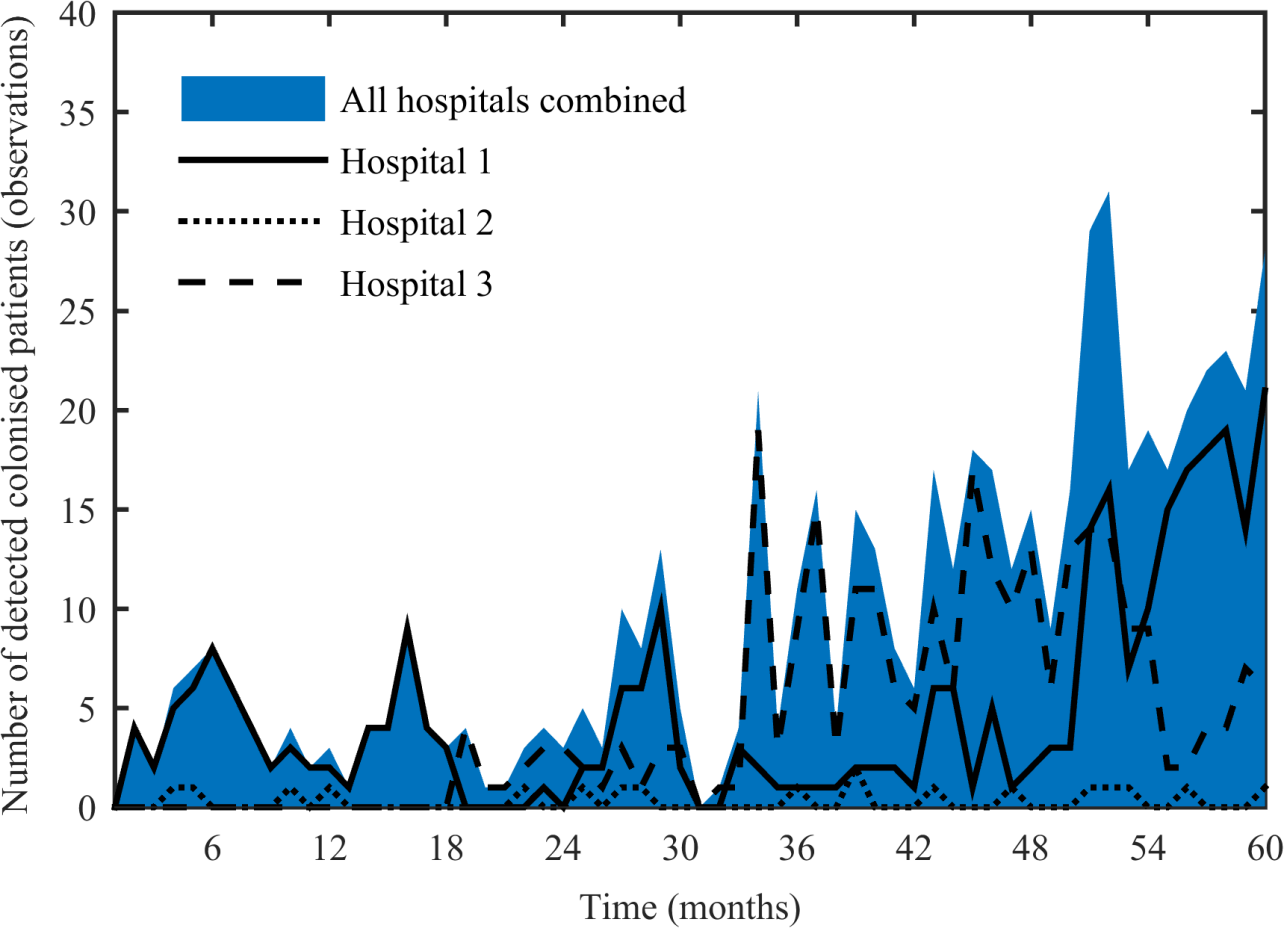
Monthly observed number of *A*. *baumannii* colonisation.

The data used in the present study were the monthly incidence rates of adult patients who were identified with *A*. *baumannii* colonisation from January 2000 to December 2004 derived from the aforementioned study (Fig 1). The mean (range) number of detected colonised patients in Hospital 1, 2 and 3 was 5 (0–21), 0 (0–2) and 6 (0–19) per month, respectively.

### Mechanistic transmission model

**Fig 2:**
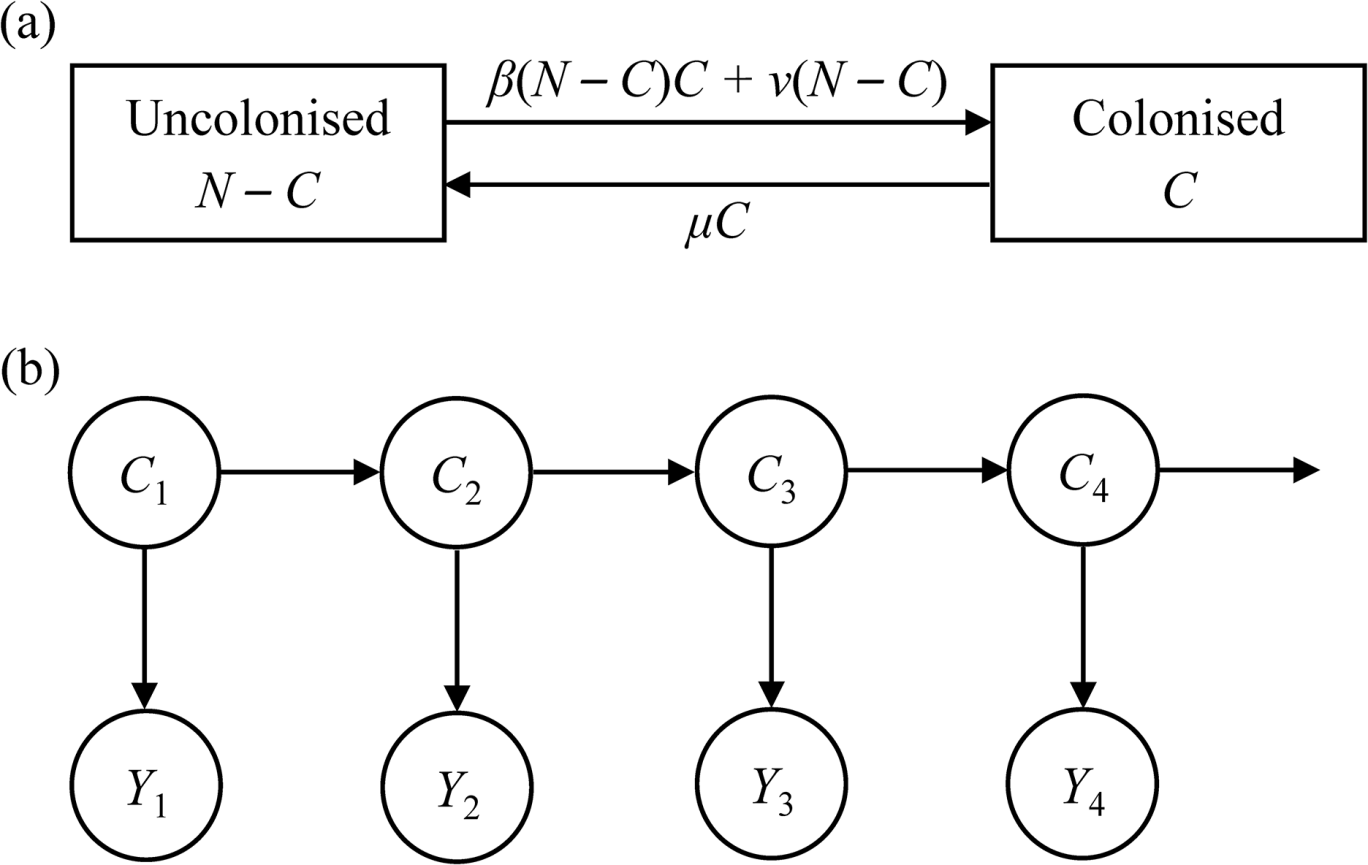
Mechanistic transmission model of *A*. *baumannii* (Fig 2A) and hidden Markov model (Fig 2B). *β*, cross-transmission coefficient; *C*, number of colonised patients; *μ*, discharge rate; *N*, ward size; *ν*, sporadic acquisition coefficient. In Fig 2B, the horizontal arrows represent the transition from one state to the next. The vertical arrows represent the probability relationship between the hidden state (*C*) and the corresponding observation (*Y*).

We used the Susceptible–Infected model structure with admission and discharge to describe the transmission dynamics of *A*. *baumannii* (Fig 2A) [13,14,22]. This model structure is a modified, more parsimonious variant of the Ross-Macdonald model in which the healthcare worker compartment in the Ross-Macdonald model is replaced by the constant, *β* × (number of colonised patients), where *β* is the transmission coefficient incorporating both direct and indirect transmission [20]. Within the model, patients were classified as being uncolonised (therefore susceptible) or colonised with *A*. *baumannii*. The number of colonised patients (both detected and undetected) is denoted by *C*. The number of uncolonised patients is *N–C*, assuming the ICU ward of fixed size (*N*) and 100% bed occupancy rate. Acquisition of *A*. *baumannii* can occur due to transmission between patients within the ward, defined as cross-transmission. This acquisition process is determined by the cross-transmission coefficient *β* (per colonised per susceptible per day) and described by the mass-action term *βC*(*N* − *C*) [14]. *A*. *baumannii* can also arise from sporadic acquisition, defined as colonisation already present at admission or any other process that is independent of the number of colonised patients such as *de novo* colonisation from patient’s gastrointestinal flora, and occurs at a rate *ν* (per susceptible per day). To put these parameters into clinical perspective, they can be converted into the average number of days required for one secondary colonised case to occur using the following equations:

The average number of days to colonisation due to cross-transmission for a susceptible individual

$$=\frac{1}{\beta C}$$ (1)

The average number of days required for one secondary colonisation arising from sporadic acquisition for the whole ICU

$$=\frac{1}{\nu (N-C)}$$ (2)

Once colonised, patients are assumed to remain so for their entire stay [14,23]. As such, the transition from colonised to uncolonised status occurs when a colonised patient is discharged from the ward and replaced by an uncolonised patient, which occurs at a rate *μ*. This discharge rate was calculated as the inverse of length of stay (LOS), which was available from our dataset. Changes in the number of colonised patients, *C*, over a small time increment, *h*, have probabilities that follow the first-order Markov process [14]. Such transition probabilities are governed by the following equations:

$$\begin{array}{l} \text{Pr}[C_{t+h}=i+1|C_{t}=i]=(N-i)\nu h+(N-i)i\beta h+o(h);\\ \text{Pr}[C_{t+h}=i-1|C_{t}=i]=\mu ih+o(h);\\ \text{Pr}[C_{t+h}=i|C_{t}=i]=1-(N-i)\nu h-(N-i)i\beta h-\mu ih+o(h);\\ \text{Pr}[C_{t+h}=j(j\neq i-1,i,i+1)|C_{t}=i]=o(h); \end{array}$$ (3)

where *o*(*h*)s are additional probabilities which we assume to have low order magnitude that can be neglected when *h* is small. The number of colonised patients (both detected and undetected), *C*, at any given time *t* is unknown, and forms a Markov process on state space *S* = {0, 1, 2, …, *N*}.

### Hidden Markov model

We aimed to estimate the cross-transmission coefficient, *β*, and the sporadic acquisition coefficient, *ν*, by fitting a structured HMM to the observed data. The HMM was used to accommodate the partially observed nature of the underlying transmission process in which, at a given time *t*, only a proportion of colonised patients is observed and therefore recorded in our dataset (i.e. observations); whereas the total number of colonised patients (both detected and undetected) is unknown (i.e. hidden). The term “structured” means this underlying continuous-time Markov chain is derived from the mechanistic transmission model as described above, in which the process of acquiring colonisation is determined by the model parameters, *β* and *ν*. This HMM framework allows the hidden states to be inferred based on the number of observations.

The HMM is illustrated in Fig 2B. It consists of the hidden states, *C*, and the number of observations, *Y*, at each time point. This observation component of the HMM consists of 60 data inputs (*Y* = *Y*_1_, *Y*_2_, …, *Y*_*n*_) of monthly incidence of *A*. *baumannii* colonisation over 5 years (*n* = 60) and a vector of time, *t* = *t*_*t*_, *t*_2_, …, *t*_*n*_, corresponding to each observation. For each observation, there is one corresponding hidden state, denoted by *C*_1_, C_2_, …, *C*_*n*_. The probability of transition from one hidden state to another is determined by the transition probability matrix and illustrated as the horizontal arrows in Fig 2B. A detailed discussion of the construction of the transition probability matrix can be found in McBryde et al. [14]. The number of hidden states at a given time *t* is conditional (probabilistically) on the corresponding number of observations at that time, and is illustrated as the vertical arrows in Fig 2B. This probability relationship is assumed to follow a binomial distribution *Y*_*k*_ ∼ *Bin*(*C*_*k*_, *d*), where *Y*_*k*_ is the number of observed colonisation at time *t*_*k*_; *C*_*k*_ is the actual number of colonisation at that time point (unobserved or hidden); and *d* is the probability of being known to be colonised given that a patient is actually colonised. Prior information on *d* is limited, except that it is between 0 and 1, and that the mean value of the sensitivity of swabbing methods reported in the literature is 70% [24,25]. We allowed for this uncertainty by randomly drawing the value for *d* from the beta (4.5, 2.5) distribution. A beta (1, 1) distribution was also assessed in sensitivity analysis. The shapes of these distributions are shown in S1 Fig

### Bayesian framework

A Bayesian framework within the HMM was used for estimating the cross-transmission coefficient, *β*, and sporadic acquisition coefficient, *ν*. Let *θ*_*p*_ = {*β*, *ν*} be the vector of model parameters. The posterior probability distributions of model parameters conditional on the dataset are given by

$$\text{Pr}(\theta_{p}|Y)\propto \pi (\theta )L(Y|\theta_{p}),$$ (4)

where *π*(*θ*) is the prior distribution of model parameters and *L*(*Y* | *θ*_*p*_) is the likelihood of the data given model parameters. Uniform *U*[0, 0.1] priors were assigned to *β* and *ν*, because little was known about these parameters except that negative values or values higher than 0.1 were biologically implausible. The likelihood function, *L*(*Y* | *θ*_*p*_), is described in McBryde et al. [14]. The posterior probability distributions were estimated using the Markov chain Monte Carlo algorithm. For each parameter, five Markov chains were constructed and run until convergence was achieved. Convergence of the Markov chains was assessed using the Gelman-Rubin method [26]. A Gelman-Rubin value of less than 1.1 was considered convergence [26]. The Markov chain Monte Carlo algorithm is comprehensively described in McBryde et al. [14]. Methodological appendices are available upon request.

The basic reproduction ratio, *R*_0_, is calculated as *β*(*N* − 1) / *μ*. It is the average number of secondary cases resulting from one single colonised individual in a totally susceptible population [27]. We also aimed to estimate the proportion of colonisation that was acquired via cross-transmission. The expected number of acquisition due to cross-transmission at time *t*_*k*+1_ following time *t*_*k*_ is *βC*_*k*_(*N* − *C*_*k*_); whereas the total number of acquisition (both cross-transmission and sporadic acquisition) is *βC*_*k*_(*N* − *C*_*k*_) + *ν*(*N* − *C*_*k*_). Therefore, the proportion of colonisation that was due to cross-transmission, *p*, is approximated by

$$p=\frac{\sum_{k=1}^{n}\beta C_{k}(N-C_{k})}{\sum_{k=1}^{n}\beta C_{k}(N-C_{k})+\nu (N-C_{k})}$$ (5)

All analysis was performed using MATLAB (version R2013b, MathWorks, Natick, MA, USA).

### Model selection

The Bayesian information criterion (BIC) was used for model selection, as previously shown to be appropriate for Bayesian HMMs [28,29]. Briefly, it is a Bayesian method for model selection, based on the trade-off between the model’s goodness of fit and the corresponding complexity of the model [28,29]. The model with the lowest BIC value is preferred [28,29]. Table 1 shows the various models that were evaluated.

**Table 1:** Comparison of different models.

Model	Estimate of *β* ×10^−4^ (95% credible interval)	Estimate of *ν* ×10^−4^ (95% credible interval)	BIC
All hospitals combined (assuming homogeneity across hospitals regarding transmission)			
One value for *β* and one value for *ν*	50 (39–71)	21 (11–39)	897
*β* = 0; one value for *ν*	0	279 (189–458)	1,284
*ν* = 0; one value for *β*	57 (46–88)	0	1,390
One value for *ν* and two values for *β* with change point at the end of month 30^a^	38 (23–54); 55 (40–90)	23 (11–53)	903
Two values for *ν* and two values for *β* with change point at the end of month 30^a^	38 (26–53); 53 (40–84)	22 (10–55); 25 (11–57)	904
Individual hospitals (assuming heterogeneity across hospitals regarding transmission)			
One value for *β* and one value for *ν* for each hospital	Hospital 1: 71 (59–95)	Hospital 1: 15 (5–36)	787
	Hospital 2: 1.7 (0.037–24)	Hospital 2: 46 (25–83)	
	Hospital 3: 36 (29–49)	Hospital 3: 4 (0.23–18)	

BIC, Bayesian information criterion; *β*, cross-transmission coefficient; *ν*, sporadic acquisition coefficient.

^a^Data set suggested a marked increase in the number of colonised patients at month 30 of the study period.

## Results

**Fig 3:**
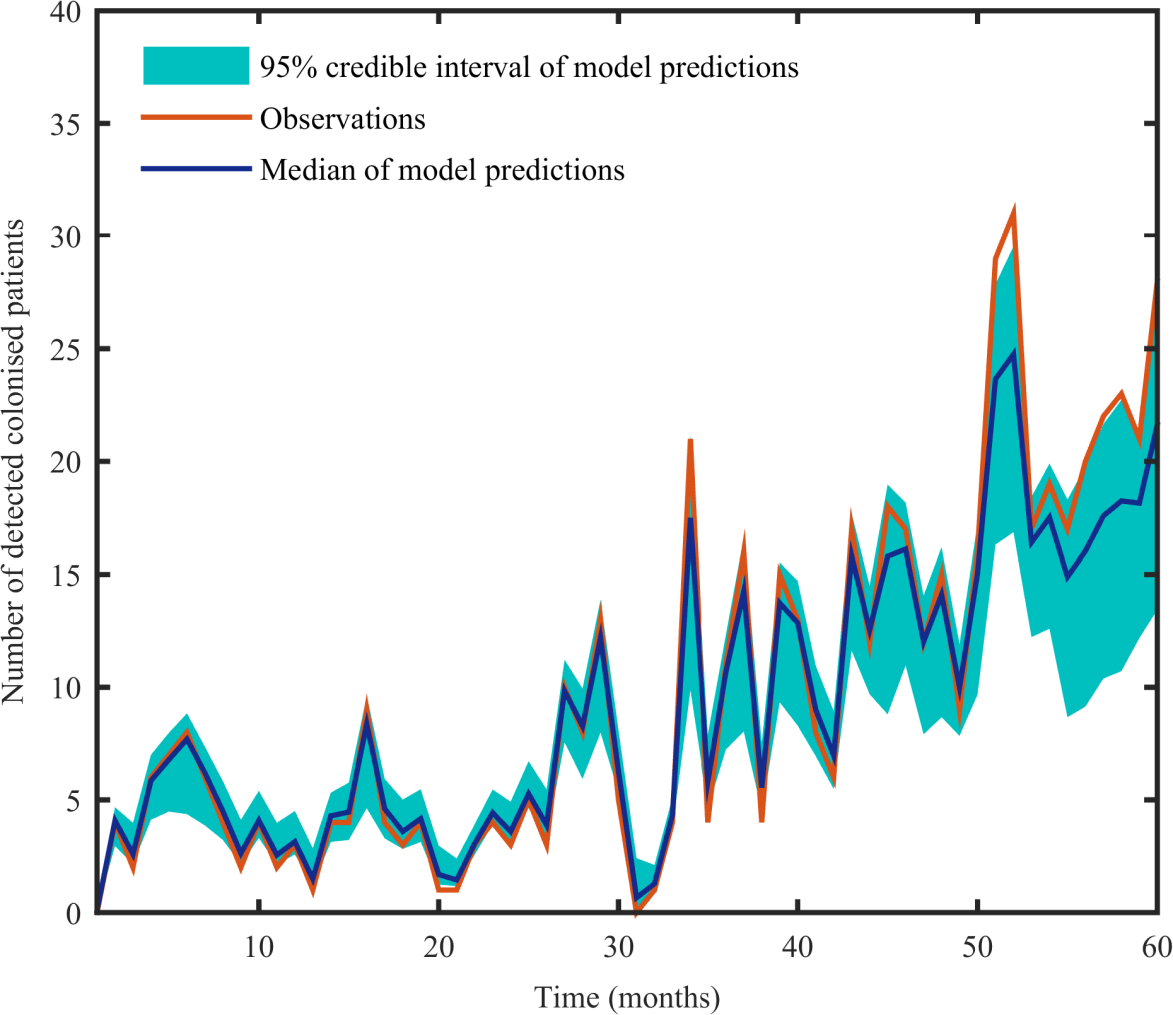
Observed number of colonised patients and predicted number of colonised patients in each month estimated by fitting the structured hidden Markov model to the observations.

The BIC values of the different models evaluated are shown in Table 1. The models with only either cross-transmission (ν = 0) or sporadic acquisition (*β* = 0) had the highest BIC values. This provides statistical support for a mixed model in which *A*. *baumannii* colonisation can be acquired via both cross-transmission and sporadically. The models that allowed for a change in transmission coefficients just prior to a marked increase in the number of colonised patients as observed in our data set (month 30, Fig 1) did not improve model fit. The model with *β* and *ν* estimated specifically for each hospital best fit the data. There is a good agreement between the observed number of colonised patients (observations) and the predicted number of detected colonised patients fitted through the HMM (predictions), further supporting that the model is appropriate for explaining the data (Fig 3).

**Fig 4:**
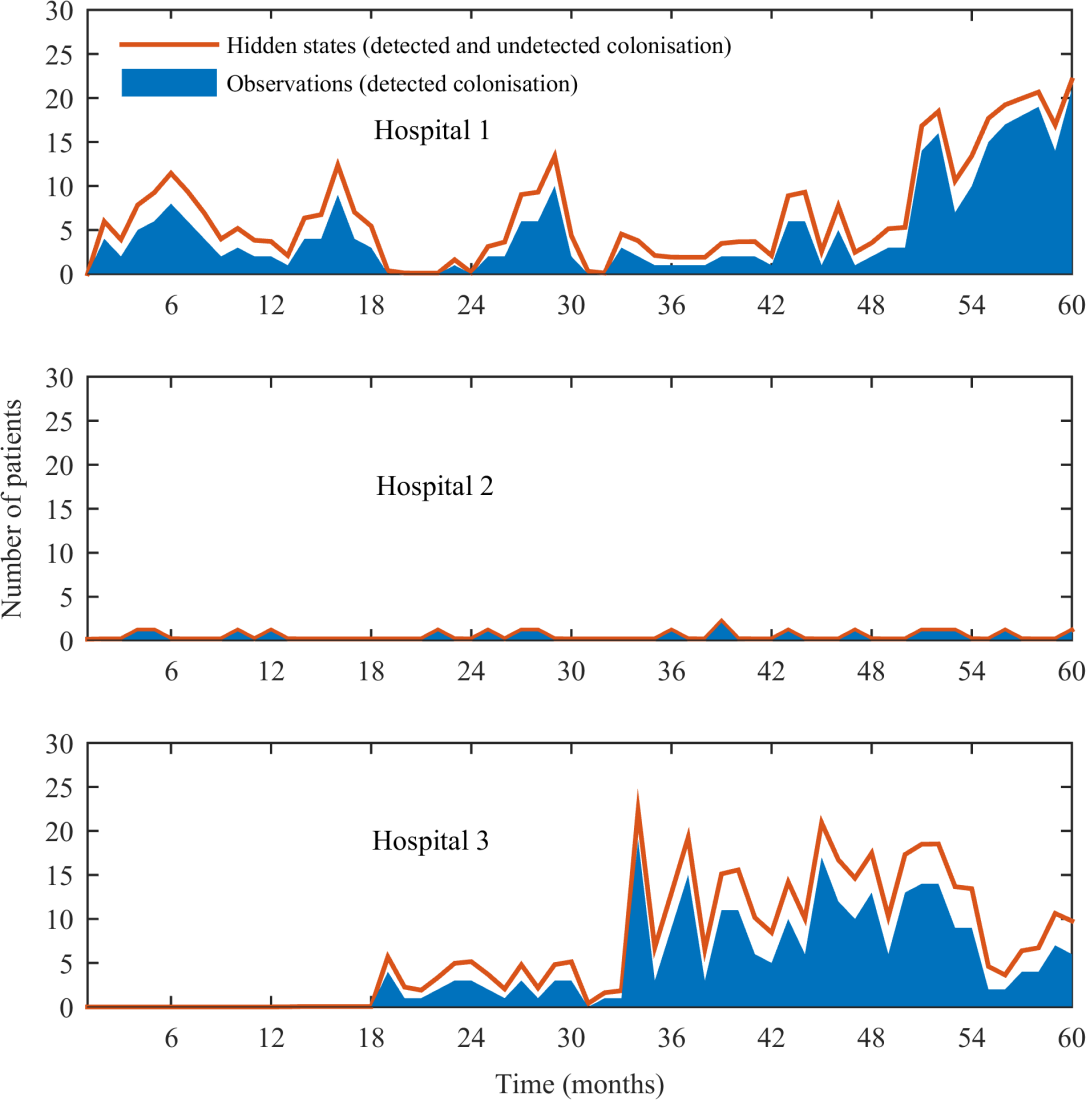
Predicted number of hidden states (detected and undetected colonised patients).

We estimated that 29% (7/24) and 28% (9/32) of patients in Hospital 1 and Hospital 3 were colonised with *A*. *baumannii* at any given time point, respectively; whereas the prevalence estimated for Hospital 2 was 4% (0.5/13) (hidden states, Fig 4). Estimates of the transmission parameters are provided in Table 2. The estimated cross-transmission coefficient, *β*, for Hospital 1, 2 and 3 was 71×10^−4^ (95% credible interval: 59–95×10^−4^), 1.7×10^−4^ (0.037–24×10^−4^), and 36×10^−4^ (29–49×10^−4^), respectively. This means that, on average, each susceptible individual in Hospital 1 and 3 will become colonised as a result of cross-transmission after 20 (95% credible interval: 15–24) days and 31 (23–38) days, respectively. We estimated that there was no cross-transmission in Hospital 2.

**Table 2:** Model parameters.

Parameters	Symbol (unit)	Value (95% credible interval)			Source
		Hospital 1	Hospital 2	Hospital 3	
Number of patients	*N* (patients)	24	13	32	Data set
Removal rate of colonised patients	*μ* (day^-1^)	0.11	0.13	0.07	Data set
Cross-transmission coefficient	*β* (×10^−4^) (/colonised/susceptible/day)	71 (59–95)	1.7 (0.037–24)	36 (29–49)	Fitted using HMM
Sporadic acquisition coefficient	*ν* (×10^−4^) (/susceptible/day)	15 (5–36)	46 (25–83)	4 (0.23–18)	Fitted using HMM
Proportion of cross-transmission	*p* (%)	96 (89–99)	1.6 (0–22)	98 (92–100)	Fitted using HMM
Basic reproduction ratio	*R*_0_	1.5 (1.2–2)	0.02 (0–0.2)	1.6 (1.3–2.2)	Fitted using HMM

HMM, hidden Markov model.

**Fig 5:**
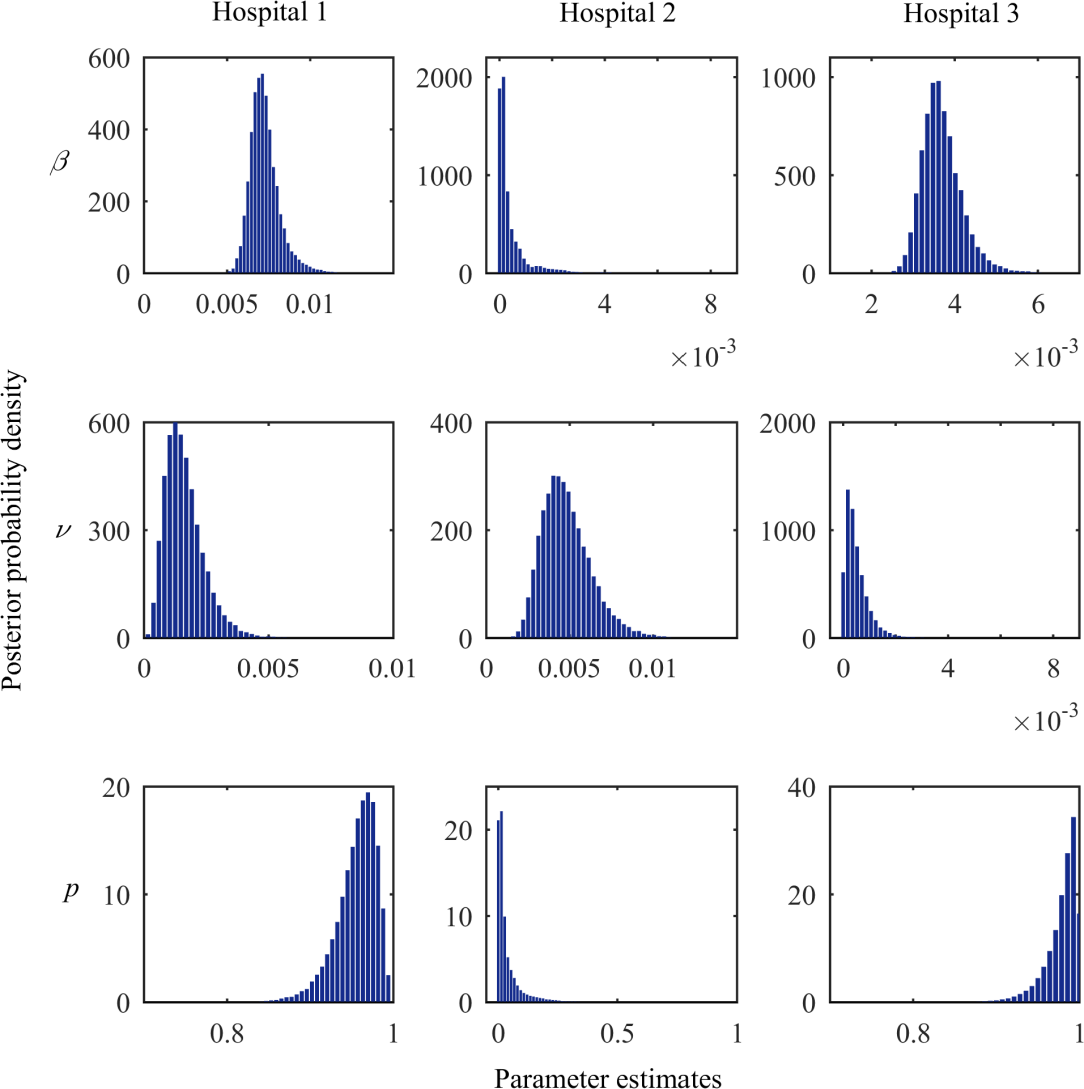
Posterior probability density of parameter estimates. Beta (4.5, 2.5) distribution was used for the probability of detection, *d*, in the observation model. *β*, cross-transmission coefficient; *ν*, sporadic acquisition coefficient; *p*, proportion of colonisation due to cross-transmission.

The estimated value for the sporadic acquisition coefficient, *ν*, for Hospital 1 was 15×10^−4^ (95% credible interval: 5–36×10^−4^). This means there is one new case due to sporadic acquisition every 39 (95% credible interval: 16–117) days for the whole ICU; whereas it only takes 17 (9–31) days to observe one new case from sporadic acquisition in Hospital 2 (*ν* = 46×10^−4^ [25–83×10^−4^]). Hospital 3 had the lowest sporadic acquisition coefficient, requiring 109 (24–1,890) days for one new case to arise from this acquisition route (*ν* = 4×10^−4^ [0.23–18×10^−4^]). The posterior probability distributions of *β* and *ν* for each hospital are shown in Fig 5. There is a weak negative correlation between *β* and *ν*. The coefficient of correlation between *β* and *ν* for Hospital 1, 2 and 3 was –0.23, –0.15 and –0.22, respectively.

**Fig 6:**
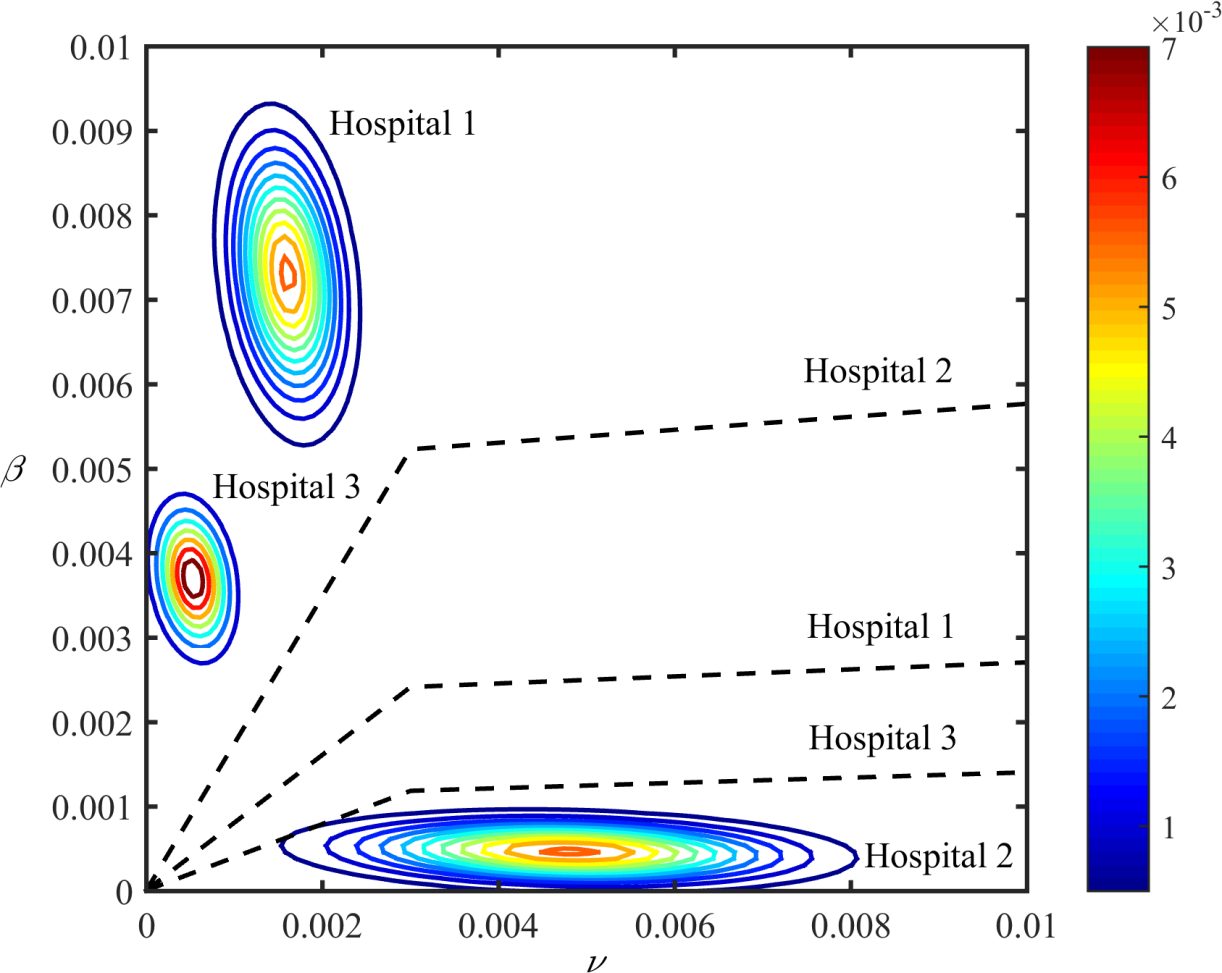
Contour plots of the likelihood of *β* (cross-transmission coefficient) and *ν* (sporadic acquisition coefficient). The colours represent different levels (as indicated in the colour bar) of the probability density of *β* and *ν* estimates. The dashed lines indicate equality between cross-transmission and sporadic acquisition (i.e. each acquisition route is responsible for 50% of colonisation). For each hospital, if the contour plot lies above the corresponding equality line, cross-transmission is a more important route of colonisation than sporadic acquisition. If the contour plot is below the corresponding equality line, sporadic acquisition is more important than cross-transmission.

Differentiating between cross-transmission and sporadic acquisition is inherently difficult because both increase the number of colonisation for a given constant number of observed acquisition. Despite this, we were able to distinguish and quantify these two sources of colonisation. By plotting the lines of equal importance of sporadic and cross-transmission colonisation (i.e. each acquisition route is responsible for 50% of colonisation), defined by *ν* = *β*×*C*_*equilibrium*_, we showed that cross-transmission was the predominant route of colonisation in Hospital 1 and 3; whereas sporadic acquisition was more important in Hospital 2 (Fig 6). Specifically, we estimated that cross-transmission was responsible for 96% (95% credible interval: 89–99%) and 98% (92–100%) of *A*. *baumannii* colonisation in Hospital 1 and 3, respectively (Table 2). In contrast, only 1.6% (0–22%) of acquisition in Hospital 2 was due to cross-transmission (Table 2). The estimated *R*_0_ for Hospital 1, 2, and 3 was 1.5 (95% credible interval: 1.2–2), 0.02 (0–0.2) and 1.6 (1.3–2.2), respectively (Table 2). There were modest changes in parameter estimates (Table 3) and their posterior distributions (S2 Fig) when a beta (1, 1) distribution was used for the probability of detection, *d*.

**Table 3:** Sensitivity of model outcomes to changes in the probability of detection, *d*.

Parameters	beta (4.5, 2.5) distribution	beta (1, 1) distribution
	*median* (*95% credible interval*)	*median* (*95% credible interval*)
Cross-transmission coefficient, *β* (×10^−4^)		
Hospital 1	71 (59–95)	83 (60–164)
Hospital 2	1.7 (0.037–24)	1.3 (0.05–38)
Hospital 3	36 (29–49)	42 (29–86)
Sporadic acquisition coefficient, *ν* (×10^−4^)		
Hospital 1	15 (5–36)	13 (3.2–43)
Hospital 2	46 (25–83)	62 (31–118)
Hospital 3	4 (0.23–18)	3 (0.2–47)
Proportion of acquisition due to cross-transmission, *p* (%)		
Hospital 1	96 (89–99)	98 (90–100)
Hospital 2	1.6 (0–22)	1.1 (0–30)
Hospital 3	98 (92–100)	99 (87–100)
Basic reproduction ratio, *R*_0_		
Hospital 1	1.5 (1.2–2)	2 (1.4–4)
Hospital 2	0.02 (0–0.2)	0.01 (0–0.38)
Hospital 3	1.6 (1.3–2.2)	1.9 (1.3–4)

## Discussion

Our study is the first to use mathematical modelling to characterise the transmission dynamics of *A*. *baumannii*. Unlike the majority of previous models [12,17–19,30,31], we incorporated sporadic acquisition, in addition to cross-transmission, to account for other potential sources of colonisation. Using an HMM with a Bayesian framework, we were able to make inferences about transmission parameters in the face of unobserved events and imperfect data. We estimated that *A*. *baumannii* can be acquired both via cross-transmission and sporadically, with the former responsible for the majority (96–98%) of colonisation in the endemic setting. While there are limited data on the transmission dynamics of *A*. *baumannii* or other Gram-negative organisms with which our results can be compared, Cooper and Lipsitch [22] and McBryde et al. [14] found that cross-transmission was the major acquisition source of Gram-positive organisms (methicillin-resistant *Staphylococcus aureus*, vancomycin-resistant enterococci) in their models. Our findings are of practical significance for setting infection control priorities in hospitals. They suggest that infection control interventions that target cross-transmission such as hand hygiene and contact precautions would have the potential to substantially reduce the spread of *A*. *baumannii*. We found that the number of secondary cases infected by one single colonised patient (*R*_0_) was above unity in Hospital 1 and 3 (1.5 and 1.6, respectively), emphasising the need for optimising infection control in these hospitals.

Several assumptions were made in our model due to the lack of data on various aspects of the transmission dynamics of the pathogen. In the observation model, we described the probability relationship between the observations and the corresponding hidden states using a binomial distribution. While other alternative observation models such as Poisson or negative binomial distributions could have been used, the binomial distribution was chosen to ensure that the number of hidden states (total number of colonised patients) is always higher than the number of observations (detected colonised patients). Indeed, using the Poisson or negative binomial distribution did not improve model fit (data not shown). Limited information is available on the probability of being known to be colonised given that a patient is actually colonised, *d*. We allowed for this uncertainty by randomly drawing the value for *d* from a beta distribution. This also allowed incorporation of the variability in detection efforts between the hospitals and over time, on which we lacked data. Nevertheless, our results are robust to changes in this variable. We assumed a constant discharge rate for each hospital, implying an exponential distribution of LOS. Our data showed that the distribution of LOS was right-skewed with a long tail and a mode close to zero, suggesting that the assumption of a constant discharge rate was plausible. We assumed that patients, once colonised, remain so until discharge. While within-ward loss of colonisation cannot be ruled out, it has been shown that colonised patients in the ICU setting typically remain so for their entire stay in the hospital [23]. In our model, changes in infection control practice were not considered because of the lack of data. Further studies are needed to quantify the effects of such changes on the transmission dynamics of *A*. *baumannii*. We assumed that uncolonised patients were equivalent with respect to susceptibility to colonisation. Future models that allow for heterogeneity in susceptibility are needed [23]. The interactions between community-acquired and hospital-acquired *A*. *baumannii* should also be considered in future studies. However, extensive clinical data would be required for such models. While we lacked genotyping data to verify our results, previous studies have shown that transmission dynamic models and genotyping approaches yielded comparable estimates [14]. Model selection for HMMs remains an unresolved methodological issue [28]. We used the BIC for model selection as it has been demonstrated to have an adequate behavior for HMMs [28,29]. Previous studies showed that the Akaike information criterion (AIC) and the deviance information criterion (DIC) are also appropriate in this setting [14,32]. Nevertheless, the results of our model selection remain unchanged when the AIC or DIC was considered (S1 Table).

Setting hospital infection control priorities is a matter of ensuring efficient allocation of scarce resources. In this regard, it is generally agreed that infection control measures should be tailored according to the importance of different acquisition routes [13]. In the absence of comprehensive epidemiological data, mathematical modelling appears to be the best alternative. Our model framework is well suited to the hospital setting and has wide applicability. It is appropriate for any hospital pathogen that can be carried asymptomatically, including other Gram-negative organisms, of which transmission data are currently lacking. The transmission parameters estimated in our study will be useful for simulation studies that require such information, for example studies considering the impact of infection control measures.

## Supporting Information

S1 Fig Shapes of beta (4.5, 2.5) and beta (1, 1) distributions for the probability of detection, *d*. (TIF)

S2 Fig Posterior probability density of parameter estimates using beta (1, 1) distribution for the probability of detection, *d*, in the observation model. *β*, cross-transmission coefficient; *ν*, sporadic acquisition coefficient; *p*, proportion of colonisation due to cross-transmission.
(TIF)

S1 Table Comparison of the results of different model selection methods (DOC)
