# Supplementary figures and images for: Characterising the Transmission Dynamics of Acinetobacter baumannii in Intensive Care Units Using Hidden Markov Models
Source: PLoS One. 2015 Jul 1;10(7):e0132037. doi: 10.1371/journal.pone.0132037 (PMC4489495; doi:10.1371/journal.pone.0132037)

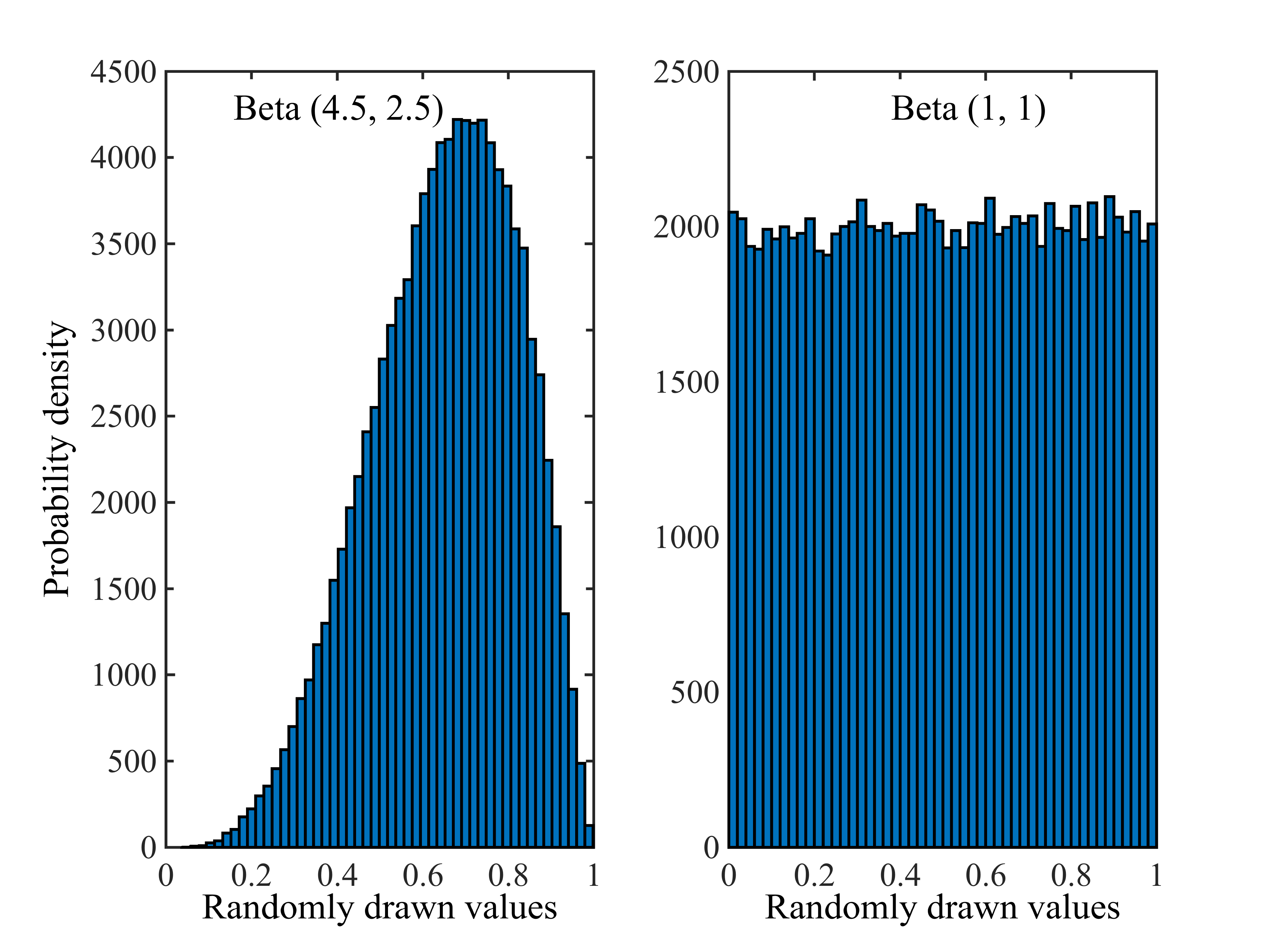

Supplement: S1 Fig — (TIF) [file pone.0132037.s001.tif]

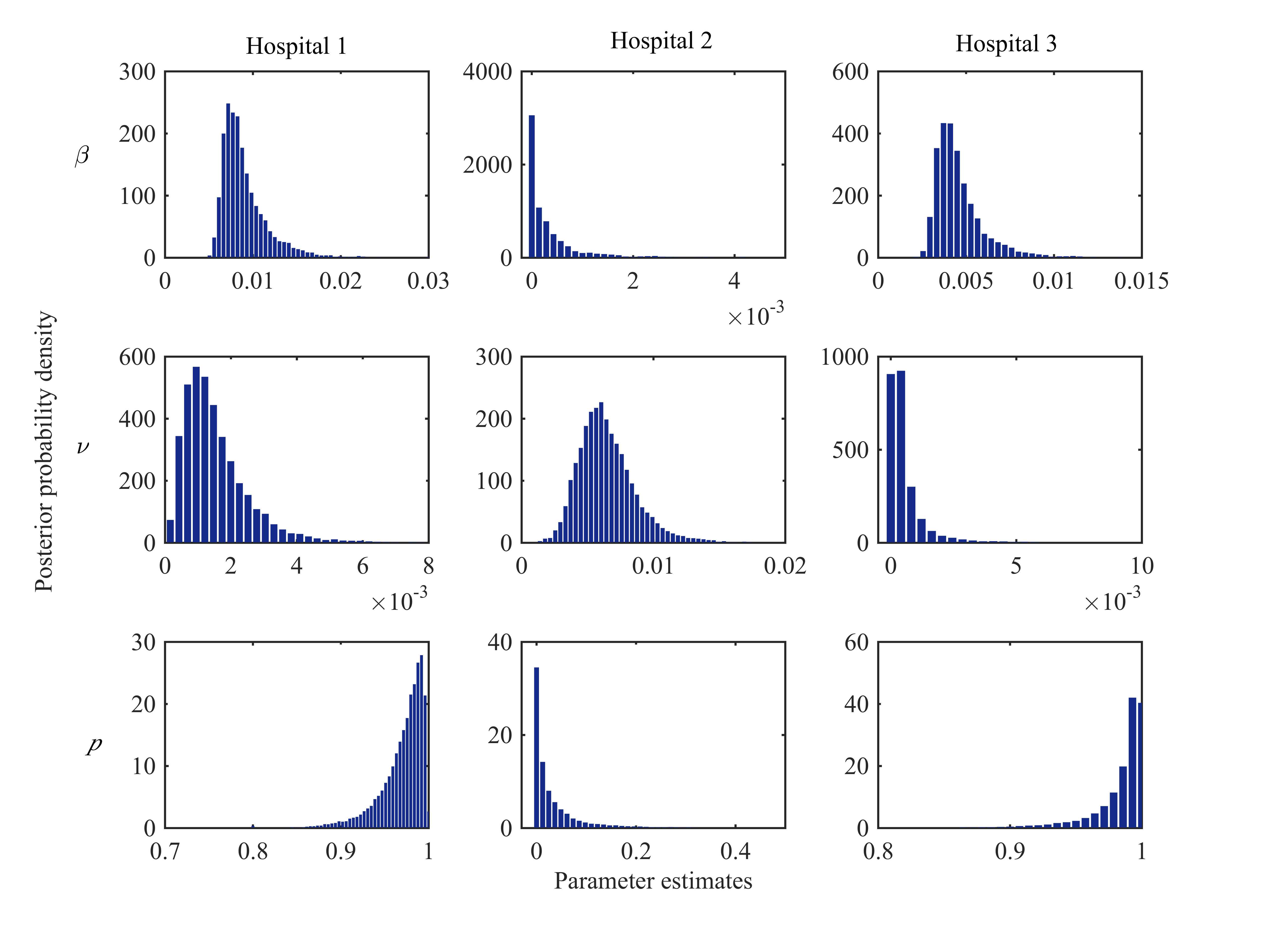

Supplement: S2 Fig — β, cross-transmission coefficient; ν, sporadic acquisition coefficient; p, proportion of colonisation due to cross-transmission. (TIF) [file pone.0132037.s002.tif]
