# Supplementary material for: Characterising the Transmission Dynamics of Acinetobacter baumannii in Intensive Care Units Using Hidden Markov Models
Source: PLoS One. 2015 Jul 1;10(7):e0132037. doi: 10.1371/journal.pone.0132037 (PMC4489495; doi:10.1371/journal.pone.0132037)
Supplement: S1 Table — (DOC) [file pone.0132037.s003.doc]

**S1 Table. Comparison of the results of different model selection methods**.

| Model | BIC | DIC | AIC |
| --- | --- | --- | --- |
| All hospitals combined (assuming homogeneity across hospitals regarding transmission) | | | |
| One value for *β* and one value for *ν* | 897 | 904 | 893 |
| *β =* 0; one value for *ν* | 1,284 | 1,302 | 1,282 |
| *ν =* 0; one value for *β* | 1,390 | 1,402 | 1,388 |
| One value for *ν* and two values for *β* with change point at the end of month 30^a^ | 903 | 909 | 897 |
| Two values for *ν* and two values for *β* with change point at the end of month 30^a^ | 904 | 909 | 898 |
| Individual hospitals (assuming heterogeneity across hospitals regarding transmission) | | | |
| One value for *β* and one value for *ν* for each hospital | 787 | 777 | 775 |

AIC, Akaike information criterion; BIC, Bayesian information criterion; *β*, cross-transmission coefficient; DIC, deviance information criterion; *ν*, sporadic acquisition coefficient.

^a^Data set suggested a marked increase in the number of colonised patients at month 30 of the study period.
